# Psychological predictors for attendance of post-HIV test counselling and linkage to care: the Umeed cohort study in Goa, India

**DOI:** 10.1186/1471-244X-14-188

**Published:** 2014-06-30

**Authors:** Rosie Mayston, Vikram Patel, Melanie Abas, Priya Korgaonkar, Ramesh Paranjape, Savio Rodrigues, Martin Prince

**Affiliations:** 1 Health Service & Population Research Department, Institute of Psychiatry, Kings College London, London SE5 8AF, UK; 2 Department of Nutrition and Public Health Intervention Research, London School of Hygiene & Tropical Medicine, London WC1E 7HT, UK; 3 Sangath, North Goa: 841/1, Near Electricity Department, Alto Porvorim, Bardez-Goa, India; 4 National AIDS Research Institute, 73, 'G'-Block, MIDC, Bhosari Pune 411 026, India; 5 Department of Microbiology, Goa Medical College and Hospital, Bambolim, Tiswadi Goa, India

**Keywords:** Linkage to care, Depression, Anxiety, HIV testing, Cognitive impairment, India

## Abstract

**Background:**

Successful linkage to care is increasingly recognised as a potentially important factor in determining the success of Antiretroviral Therapy treatment programmes. However, the role of psychological factors during the early part of the continuum of care has so far been under-investigated. The objective of the Umeed study was to evaluate the impact of Common Mental Disorder (CMD), hazardous alcohol use and low cognitive functioning upon attendance for post-test counselling and linkage to care among people attending for HIV-testing in Goa, India.

**Methods:**

The study was a prospective cohort design. Participants were recruited at the time of attending for testing and were asked to complete a baseline interview covering sociodemographic characteristics and mental health exposures. HIV status, post-test counselling (PTC) and Antiretroviral Treatment (ART) Centre data were extracted from clinical records.

**Results:**

Among 1934 participants, CMD predicted non-attendance for PTC (adjusted OR = 0.51, 0.21-0.82). There was tentative evidence of an association between hazardous alcohol use and non-attendance for PTC (adjusted OR = 0.69, 0.45-1.02). There was no evidence of an association between CMD caseness and attendance for ART. However, post-hoc analyses showed an association between increasing symptoms of CMD and non-attendance.

**Conclusions:**

Although participation rates were high (86%), non-participation was a possible source of bias. Cognitive tests had not been previously validated in a young population in Goa. The context in which cognitive testing took place may have contributed to the high prevalence of low scores. Findings suggest the need to move towards a broader conceptualisation of the interrelationship between mental health and HIV. It may be important to consider the impact of symptoms of depression and anxiety at every stage of the continuum of care, including immediately after diagnosis and when initiating contact with treatment services.

## Background

Timely presentation at services and minimisation of delays in initiation of treatment have been identified as potentially important contributory factors to reducing the high levels of early mortality observed in treatment programmes for HIV/AIDS [1,2]. Recent research carried out among people living with HIV/AIDS has led to wider recognition of the important contribution of mental disorder, in particular, depression, to HIV clinical outcomes. People living with HIV have a high prevalence of depression and other common mental disorders (CMD), alcohol use disorders, and neurocognitive impairment [3,4]. These conditions are associated with poor adherence to antiretroviral therapy [5-7] disease progression and mortality [8]. However, we know very little about the impact of adverse mental health on engagement with services early in the continuum of care for HIV/AIDS.

The mental health of people coming for HIV testing has been assessed in just three low or middle income country (LMIC) studies [9-11]. Only one, from South Africa, examined the association of mental health with an aspect of linkage to care [11]. Authors found that depression was independently associated with failing to obtain a CD4 count (after being referred for testing by a healthcare provider) (RR = 0 · 82, 0 · 72-0 · 94). No other studies were identified that specifically examined the impact of either CMD or neurocognitive impairment upon attendance for post-test counselling, or linkage to care after diagnosis.

The aim of the “Umeed” study, carried out in Goa, India was to investigate the independent impact of symptoms of CMD, hazardous and dependent alcohol use and cognitive impairment on two early outcomes in the pathway to treatment: attendance both for post-test counselling and initial presentation at treatment services after diagnosis and referral (linkage to care). (“Umeed” means “hope” in Hindi and was chosen as an appropriate study name by team members).

## Methods

### Study design

The “Umeed” project was a prospective cohort study. Participants were recruited at the time of attending for pre-test counselling and testing at Goa Medical College. Structured baseline interviews were carried out by research assistants on-site, at the time of this first visit. Follow-up data on HIV status, attendance for post-test counselling, and (for those found to be HIV positive) attendance at an ART treatment centre for initial assessment was obtained through data linkage to routine clinical records.

### Setting and participants

Goa is an Indian state with a population of 1 · 34 million (Government of Goa, 2010). For the purposes of HIV/AIDS surveillance, Goa is divided into two districts: the north is one of India’s high prevalence districts, with more than one percent of women testing positive at antenatal care while the south is medium prevalence, with a high level of HIV/AIDS among high-risk groups (more than five percent prevalence among those attending STI clinics) (UNGASS, 2008). The cohort was recruited from attendees of the Integrated Counselling and Testing Centre (ICTC) at Goa Medical College (GMC), which serves both the north and south districts and is the largest public testing centre in the state, conducting seventy percent of tests in 2006 (n = 7664). Participants were recruited between January 2008 and January 2010 and followed-up until March 2010.

### Participant recruitment

Participants were recruited at the time of attending the ICTC for pre-test counselling and testing, as “walk-in” clients (self-referrals, or primary care physician referrals) or those formally referred for testing by doctors from other GMC departments. Individuals whose purpose for attending the ICTC was to undertake pre-test counselling and a HIV blood test were eligible to take part. Other inclusion criteria were fluency in Konkani, Hindi, or English and a lower age limit of 18 years. Written informed consent for participation and data linkage with test results was sought from all potential participants. (A thumb print to indicate consent and the signature of an independent witness was sought from those unable to sign their names due to low literacy). Questionnaires were extensively piloted among people coming for testing for HIV in Goa to ensure that questions included in the final version of the questionnaire were well understood among this population. The study was approved by local and international research ethics committees (Sangath, Indian Council for Medical Research and King’s College London).

### Measures

Questions relating to sociodemographic characteristics, HIV-related behaviours, beliefs and knowledge (transmission and prevention knowledge, disclosure plans, sexual behaviour, knowledge and symptoms of sexually transmitted infections) and a screening assessment for mental, substance and alcohol use disorders and cognitive testing were included in the baseline interviews. Basic sociodemographic data were collected from those who declined to participate.

Common Mental Disorder (CMD): To identify clinically relevant symptoms of depression, anxiety and panic, components of the Patient Health Questionnaire, comprising the brief PHQ-9, the seven item Generalised Anxiety Disorder scale (GAD-7), and the panic disorder module [12] were included in the baseline questionnaire. The PHQ-9 has been previously validated in Goa for the detection of CMD in primary care and was one of five instruments to achieve an area under the curve (AUC) from receiver operating characteristic analysis of at least 0 · 80 (0 · 84 for the PHQ-9) [13]. In the Umeed study, as recommended by Spitzer et al. [12], a cut-off of ten was used as an indicator of clinically relevant major depression. A cut-off of ten for the GAD 7 was used to define clinically relevant generalised anxiety [14]. Participants reporting “yes” to each of the first four questions relating to panic attacks were considered probable panic disorder cases. At the time of conducting the study, there were no measures of anxiety symptoms that had been validated in a local HIV-affected population. Therefore, we decided to use brief measures and recommended cut-offs that showed good validity in primary care and outpatient populations elsewhere [15]. Participants thus identified as having symptoms, to a clinically significant degree, of either major depression or generalised anxiety disorder or panic disorder were categorised as screening positive for a common mental disorder. The rationale for this approach is the high level of comorbidity between these disorders, their close underlying nosological relationship [16] and the likely common mechanisms (psychological distress, disability and negative cognitions) affecting HIV service engagement. It is important to note that the cut-points applied establish a high probability of having the relevant disorders, but do not constitute a clinical diagnosis.

Alcohol Use Disorder (AUD): The AUDIT is a ten item questionnaire designed to screen for hazardous and harmful drinking (WHO, 2001), assessing consumption, dependence and alcohol related problems (WHO, 2001). The instrument has been widely used in India [17] including Goa [18]. A recent validity study in Goa demonstrated good sensitivity and specificity, with an area under the curve of 0 · 87 when compared to alcohol abuse and dependency criteria. We used the lower cut-point of eight or above, validated in most primary care studies as the optimal threshold for identifying hazardous or harmful drinking and used in US national alcohol surveys [19,20]. These individuals (including hazardous and dependent drinkers) are subsequently referred to as screening positive for an alcohol use disorder.

Cognitive impairment: The two measures of cognitive functioning included were word list learning (assessing memory) and animal naming (measuring verbal fluency, an aspect of executive functioning, known to be a common deficit in people living with HIV/AIDS [21]. Interviewers read out a ten word list (adapted for use in India [22]) three times. Participants were asked to recall the list of words immediately after each reading and finally requested to recall words after a delay of several minutes in which another section of the questionnaire was completed. This final delayed recall total out of ten was recorded. For the animal naming test, participants were asked to tell the interviewer as many different types of animals as they could recall during one minute. These tests originate from the Consortium to Establish a Registry of Alzheimer’s Disease (CERAD) test battery and have been validated among older people in Goa as part of the development of a cross-cultural diagnostic instrument for dementia [23]. Education-specific norms (1 · 5 standard deviations below the mean) for the youngest age-group studied (60–64 years) were used to indicate likely cognitive impairment.

### Outcome measures

**Figure 1:**
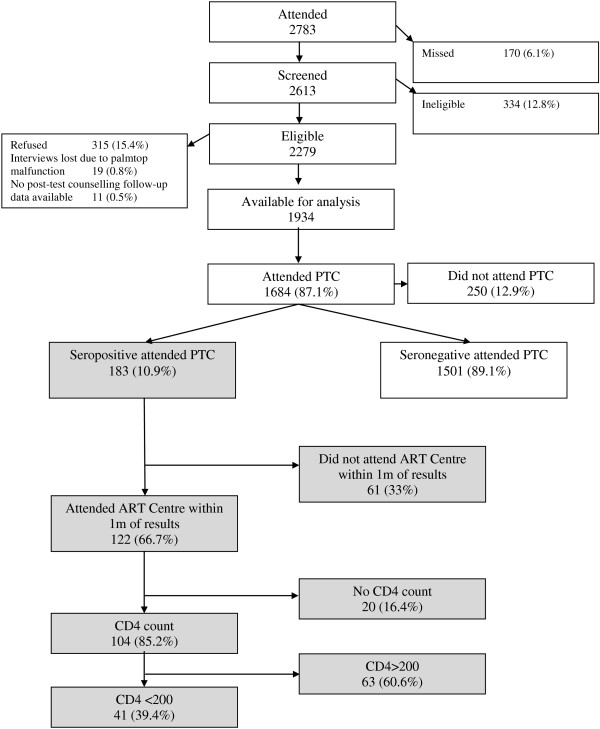
Pathways through HIV services.

At pre-test counselling, service-users were requested to return for test results after one week. At post-test counselling, those who received a positive test result were advised to attend the ART Centre (at the same hospital) as soon as possible in order to undergo further assessment of disease status and treatment eligibility.Outcome data were collected from routine clinical records maintained by ICTC and ART Centre staff by an Umeed team member not involved in baseline data collection. HIV status and date of attendance for post-test counselling (if attended) were extracted from ICTC records and date of first attendance of the ART Treatment Centre, date of attendance for CD4 count and CD4 count (for those who attended) were extracted from ART Centre records. Study participants records were identified using the unique identifier assigned to participants by counsellors at pre-test counselling. Record-keeping is closely monitored by the Head of Microbiology. The high quality is demonstrated by the low proportion of study participants for whom no data on attendance for post-test counselling was available (0 · 48%, see Figure 1).

A minimum of one month follow-up after attendance for post-test counselling was carried out for all HIV-positive participants who had attended for post-test counselling. Follow-up for three months was obtained for 152 (83%); follow-up for six months was obtained for 137 (75%). Whether participants had attended within 30 days of post-test counselling was used as the cut-off point for determining attendance/non-attendance at the ART Centre.

### Data analysis

Chi-squared tests were used to compare the sociodemographic characteristics of participants with the characteristics of those who refused to take part in Umeed.

Our hypotheses were: a) that CMD, AUD and cognitive impairment, assessed at attendance for HIV-testing, would be independently associated with non-attendance for post-test counselling; b) that the same exposures would be independently associated with non-attendance at the antiretroviral (ART) treatment centre (among those receiving a HIV diagnosis). In the testing of both hypotheses, we moved from bivariate (chi-squared tests for homogeneity and crude odds ratios) to multivariate analyses (logistic regression), adjusting for potential confounders. Criteria for inclusion in final models were: a) variables considered a priori confounders (for example, association of impaired cognitive functioning with AUD and CMD); or b) variables selected using a “change in estimate” method ie. those that resulted in a significant change in the effect of exposure of interest upon outcome (more than 10 percent change in odds ratio). We were interested to see whether HIV status had a modifying effect upon any observed associations between psychological variables and attendance outcomes as differential impact by HIV status has potentially important implications for service providers and policymakers. In the test of the first hypothesis we introduced an interaction term (psychological exposure by HIV status) as a sensitivity analysis in the final stage of modelling. Due to the negligible prevalence of AUD among women (0 · 9 percent), all analyses of alcohol use were carried out using a men-only sample (n = 915). To explore the independent effects of depression and anxiety symptoms upon attendance of the ART Centre and attendance for post-test counselling, post-hoc analyses were carried out. PHQ-9 and GAD-7 scores were divided into four categories (0, 1–4, 5–9, >10 points). In order to test the associations between depression and anxiety symptom categories and each of the attendance outcomes, we used the same approach as that used in the testing of the a priori hypotheses.

## Results

### Sample characteristics

2783 participants attended the ICTC for pre-test counselling during the study period. Of the 2613 attendees screened for Umeed, 2279 were deemed eligible to take part in the study. Of these, 315 (13 · 8%) declined to participate, hence 1934 were enrolled in Umeed (see Figure 1). Those who refused were more likely to originate from Goa, to be Christian as opposed to Hindu or Muslim, and to have lower levels of education (Additional file 1: Table S1).

See Table 1 for sociodemographic characteristics of the sample. The prevalence of any CMD was 5 · 4 percent. Depression was the most common CMD disorder- of those with any CMD, 80 percent scored 10 or more on the PHQ-9. 13 · 3 percent of participants screened positive for AUD (27 percent among men). Around a quarter of the sample scored below the educational norm on two cognitive tests of delayed recall (23 · 1 percent) and verbal fluency (25 · 9 percent). Prevalence of non-prescribed drug use was very low (1 · 3 percent) (not presented in table). Eleven percent of the sample tested seropositive for HIV.

**Table 1:** Bivariate analysis of association of mental health and demographic variables with attendance of post-test counselling

**Variable**	**Prevalence among total sample N (%)**		**Attendance of PTC among exposure category N (%)**		**Unadjusted OR with 95% confidence interval**		**P-value**
Total	1934	(100.00)	1684	(87.07)			
Symptoms of common mental disorder	104	(5.38)	80	(76.92)	0.47	(0.29-0.76)	<0.01
Hazardous alcohol use (men only)	257	(13.29)	212	(82.49)	0.66	(0.46-0.93)	0.02
Cognitive functioning							
Delayed recall: scored below educational norm	466	(23.06)	380	(85.20)	1.31	(0.99-1.75)	0.06
Verbal fluency: scored below educational norm	500	(25.85)	429	(85.80)	0.81	(0.60-1.10)	0.18
Male	915	(47.31)	784	(81.75)	1.00		
Female	1019	(52.69)	900	(85.87)	1.36	(1.06-1.73)	0.01
Age						Overall test for trend	0.04
18-25	444	(22.96)	371	(83.56)	1.00		
26-30	368	(19.03)	325	(88.32)	1.49	(0.99-2.23)	0.06
31-34	307	(15.87)	266	(86.64)	1.28	(0.84-1.93)	0.25
35-40	299	(15.46)	266	(88.96)	1.59	(1.02-2.46)	0.04
41-45	168	(8.69)	145	(86.31)	1.24	(0.75-2.06)	0.40
46-50	155	(8.01)	141	(90.97)	1.98	(1.08-3.62)	0.03
50+	193	(9.98)	170	(88.08)	1.45	(0.88-2.40)	0.14
Religion						Overall test for heterogeneity	0.60
Hindu	1406	72.70	1223	(86.98)	1.00		
Christian	316	16.34	275	(87.03)	1.00	(0.70-1.44)	0.98
Muslim	209	10.83	184	(88.04)	1.10	(0.71-1.72)	0.67
Other	3	0.15	2	(66.67)	0.30	(0.03-3.32)	0.33
Marital status						Overall test for heterogeneity	0.01
Married/co-habiting	1351	(69.86)	1195	(88.45)	1.00		
Widowed	16	(0.83)	16	(100.00)	-----	------	0.15
Separated/divorced	138	(7.14)	117	(84.78)	0.73	(0.44-1.19)	0.21
Never married	429	(22.18)	356	(82.98)	0.64	(0.47-0.86)	<0.01
Education						Overall test for trend	0.50
None	350	(18.10)	303	(86.57)	1.00		
Primary school	485	(25.08)	429	(88.45)	1.19	(0.78-1.80)	0.42
Secondary school	941	(48.66)	820	(87.14)	1.05	(0.73-1.51)	0.79
Further education	158	(8.17)	132	(83.54)	0.79	(0.47-1.33)	0.37
Employment							
Paid employee	998	(51.60)	858	(85.97)	1.00	Overall test for heterogeneity	0.09
Student	50	(2.59)	38	(76.00)	0.52	(0.26-1.01)	0.06
Seasonal work	44	(2.28)	38	(86.36)	1.03	(0.43-2.49)	0.94
Self-employed: small-scale business	58	(3.00)	53	(91.38)	1.73	(0.68-4.40)	0.25
Owns business	107	(5.53)	92	(85.98)	1.00	(0.56-1.78)	1.00
Unemployed	243	(12.56)	218	(89.71)	1.42	(0.91-2.23)	0.13
No work outside home	434	(22.44)	387	(89.17)	1.34	(0.95-1.91)	0.10
Perceived likelihood of testing positive for HIV							
Likely/very likely	85	(4.40)	70	(82.35)	0.68	(0.38-1.21)	0.18
HIV status							
HIV-positive	221	(11.43)	183	(82.81)	0.68	(0.47-0.99)	0.05

### Pathway through services

Of those tested, 1,684 (87 · 1%) attended post-test counselling, of whom 183 (10 · 9%) were seropositive. 250 did not attend post-test counselling, of whom 38 (15 · 2%) were seropositive. Of the 183 individuals who received their positive test results, and were referred to the ART Centre for initial assessment, 122 (66 · 7%) attended within one month of receiving test results. Only two participants attended the ART Centre after 30 days.

### Mental health and attendance for post-test counselling

Attendance for post-test counselling was lower among those screening positive for CMD (Table 1) (crude odds ratio [OR] 0 · 47, 95% CI 0 · 29-0 · 76, and for those screening positive for AUD (OR 0 · 67, 95% CI 0 · 45-0 · 99). Low cognitive function (as measured by either test) was not found to be associated with attendance for post-test counselling. Those testing positive for HIV (OR 0 · 68, 95% CI 0 · 47-0 · 99), younger clients, men, and those not currently married were also less likely to attend.

After controlling for sex, HIV status, cognitive impairment and AUD, the association between screening positive for CMD and non-attendance for post-test counselling was little changed (Adjusted OR 0 · 51, 95% CI 0 · 30-0 · 82). After adjusting for the effects of sex, employment status, HIV status, CMD and cognitive functioning; the association between screening positive for AUD (AOR 0 · 69, 95% CI 0 · 45-1 · 03), was little changed, but no longer statistically significant. Although the association of CMD and AUD upon attendance for post-test counselling was larger among seropositive than seronegative participants, there was no evidence for effect modification of the association with CMD (Model 4, Table 2) or AUD (Model 8, Table 2).

**Table 2:** Multivariate analysis of association of mental health problems with attendance of post-test counselling

**Model**	**Variables**	**OR with 95% confidence interval**		**P-value**
Analysis of association of CMD with attendance of post-test counselling				
1	CMD	0.47	0.29-0.76	<0.01
2	CMD + sex	0.47	0.29-0.75	<0.01
3	Model 2 + HIV status + AUD + impaired memory + impaired animal naming	0.51	0.31-0.82	<0.01
4	Model 3 + interaction term: HIV status x CMD	0.72	0.23-2.23	0.56
	Interaction term	0.40	0.15-1.07	0.07
	Effect of CMD (in HIV + ve)			
Analysis of association of AUD with attendance of post-test counselling (men only)				
5	AUD	0.67	0.45-0.99	0.05
6	AUD + age + employment	0.63	0.42-0.95	0.03
7	Model 6 + HIV status + CMD + impaired memory + impaired animal naming.	0.69	0.45-1.03	0.07
8	Model 7 + interaction term: HIV status x AUD	0.54	0.17-1.66	0.28
	Interaction term	0.40	0.14-1.15	0.09
	Effect of AUD (in HIV + ve)			

### Associations with linkage to care

Among those people who tested positive for HIV and were therefore eligible for ART, bivariate analyses showed no relationships between CMD or cognitive impairment and attendance for such treatment (Table 3).

**Table 3:** Bivariate analysis of association of mental health and demographic variables with attendance at ART Centre within one month of testing

**Variable**	**Prevalence among those attending post-test counselling and receiving a positive HIV test N (%)**		**Attendance at ART Centre among exposure categories N (%)**		**Unadjusted OR with 95% confidence interval**		**P-value**
Total	183		122	66.67			
Common mental disorder	14	7.65	9	64.29	0.74	0.23-2.36	0.62
AUD (men only)	31	33.33	26	83.87	1.96	0.64-6.04	0.23
Cognitive functioning							
Delayed recall. Scored below educational norm	78	42.62	54	69.23	0.82	0.44-1.53	0.53
Verbal fluency. Scored below educational norm	55	30.05	39	70.91	0.76	0.38-1.50	0.43
Male	93	50.82	71	76.34			
Female	90	49.18	51	56.67	0.41	0.21-0.76	0.01
Age							
Less than 35 years	88	48.09	51	57.95			
More than 35 years	95	51.91	71	74.74	2.15	1.13-4.07	0.02
Religion							
Hindu	123	67.96	81	65.85			
Christian	43	23.26	28	65.12	2.07	0.55-7.76	0.93
Muslim	15	8.29	12	80.00	0.97	0.47-2.01	0.78
Marital status							
Married/co-habiting	131	71.58	95	72.52			
Not currently married	52	28.42	27	51.92	0.77	0.63-0.94	0.01
Education							
Up to primary	99	54.10	65	65.66			
Secondary school and above	84	45.90	57	67.86	1.10	0.59-2.05	0.75
Employment							
Economically active	1120	65.57	86	71.67			
Not earning money	63	34.43	36	57.14	0.53	0.28-1.01	0.05
Perceived likelihood of testing positive for HIV							
Likely/very likely	53	28.96	35	66.04	1.24	0.57-2.70	0.58

### Post-hoc analyses

We carried out exploratory analyses to examine associations between PHQ-9 and GAD-7 score categories and ART Centre attendance, obtaining a CD4 count, having a low CD4 count (<200/ml^3^), attendance of post-test counselling. Exploratory analyses suggested an association between depressive symptoms (PHQ-9 score) and attendance at the ART Centrethat was robust to controlling for the effects of sex, age, marital status and employment, but with reduced odds of attendance among all groups scoring any depression symptoms (Additional file 1: Table S2). The odds of attendance declined monotonically with increasing numbers of depression and anxiety symptoms regardless of adjustment for potential confounders (Additional file 1: Table S2). Neither PHQ-9 score category nor GAD-7 score category were found to be associated with obtaining a CD4 count (p = 0 · 21; p = 0 · 06, respectively) or obtaining a low count (<200/ml^3^) (p = 0 · 25; p = 0 · 20, respectively).

When the same exposure definitions were tested for the association with attendance for post-test counselling, reduced odds of attendance seemed to be concentrated mainly or exclusively among those scoring 10 or more points (hence meeting the criteria to screen positive for a CMD), before and after controlling for sex, HIV status, hazardous alcohol use, verbal fluency and delayed recall (Additional file 1: Table S3).

## Discussion

The Umeed cohort is one of the first prospective studies to examine the effects of psychological factors upon access to services during the important early stages of the continuum of care for HIV/AIDS. We hypothesised that screening positive for CMD or AUD, and having low scores on test of memory or verbal fluency would independently predict non-attendance for post-test counselling, and non-contact with the ART Centre (among those found to be seropositive for HIV). Those screening positive for any CMD were around half as likely to attend for post-test counselling. We also identified weaker evidence for an association between screening positive for alcohol use disorder and attendance for post-test counselling (AOR = 0 · 69, 0 · 45-1 · 03). CMD and alcohol use disorder had similar effects upon attendance for post-test counselling across HIV-positive and negative groups. Among the smaller sample of HIV-infected participants who had attended for post-test counselling (n = 183), planned analyses revealed no evidence of any association between CMD and attendance at the ART Centre. However, exploratory post-hoc analyses showed that people with symptoms of depression and anxiety were much less likely to attend the ART Centre compared to those with no symptoms. Symptoms of depression and anxiety were not associated with either obtaining a CD4 count or having a low CD4 count (where one was obtained). We found no evidence of any association between cognitive impairment and engagement with HIV services.

Determinants of receipt of diagnosis among those undergoing testing are under-researched. Studies in low and middle income settings have tended to focus upon demographic and HIV-related correlates of attendance [24-27]. The only studies to examine the effects of psychological factors upon attendance for post-test counselling were carried out in high income settings and focused mainly on substance use [28-30]. In a sample of people with severe mental illness no association was found between baseline psychiatric diagnostic category and attendance for post-test counselling [28].

Our post-hoc analyses suggest that it is important to consider the broader impact of symptoms of anxiety and depression upon access to care for HIV/AIDS beyond the clinically relevant diagnostic categories. Risk for non-attendance for PTC was concentrated among the 5 · 4% with clinically significant CMD, nearly a quarter of whom did not re-attend. However, among those who received a HIV diagnosis, attendance for assessment at the ART was substantially reduced among all groups with any anxiety or depression symptoms. Three-quarters of those referred to the ART Centre had some symptoms and more than a third of those with symptoms did not attend. The mechanism for these different risk thresholds could not be clarified but the impact of receiving a positive test result may be contingent upon pre-test mental state, together with other post-diagnostic factors that might lead to an increase in symptoms in this group, and/or unresolved anger, guilt, loss and denial [31,32]. These possibilities warrant further investigation. It will also be important to test the effectiveness of targeted intervention during and after post-test counselling with the aim of increasing linkage to care.

Currently, there are few studies with which these post-hoc findings may be compared. In a US sample of people who had recently been diagnosed with HIV, depression was a borderline statistically significant predictor of not attending HIV treatment services within three months of follow-up (AOR = 2 · 00, 95% CI = 0 · 96-4 · 14) [33]. The small amount of research on the relationship between mental health and linkage to care in low income settings has tended to focus on alcohol use rather than common mental disorder. In the STIAL study, a brief measure of psychological distress (the MHI-5), was associated with a small decreased risk in linkage to care (defined as obtaining a CD4 count, conditional upon registering at an ART centre). The threshold applied to the MHI-5 identified 55% of ART attendees as having psychological morbidity, suggesting that many would have been subclinical cases. Therefore, while caution is indicated in making inferences from post-hoc findings, there is some independent evidence that subclinical psychological morbidity may have a negative impact on linkage to care. Also, it should be noted that the associations in the US study and the STIAL study were for different segments of the care pathway; in the current study mental health was measured before diagnosis (the US study baseline assessment was within 90 days of diagnosis). Unlike STIAL, we found no association between depression or anxiety symptoms and obtaining a CD4 count, but, due to progressive attrition these analyses were underpowered.

### Limitations

Non-participation was a possible source of bias in Umeed: although 86% of those who were screened eligible agreed to take part, being Goan, having attained primary school education only and being Christian were all associated with refusal. Ethical considerations meant that it was not possible to obtain outcome data on people who had declined to participate. Although none of the demographic factors found to be associated with participation were associated with attendance outcomes, it cannot be guaranteed that bias did not arise. As clinic attendees who were “missed” for screening for eligibility -(usually at busy times in the clinic when all researchers were engaged with other participants) were likely to be missed at random, we would not expect those “missed” to bias our results.

Another potential limitation of the Umeed study is that the cognitive tests had not previously been used or validated in a younger adult Goan population, and education specific norms were derived from the youngest (60–64 year) age group from a previous study conducted in Goa and other sites in India [23]. The high prevalence of apparent low cognitive functioning among Umeed participants is surprising given their broader and mainly younger age distribution. The test context (environmental distractions, and anxiety regarding the HIV test) may have impaired concentration and hence performance. The higher prevalence of impaired verbal fluency, but not memory impairment, among those who were HIV seropositive [34] was consistent with previous research [35] and supports construct validity. The fact that the high levels of cognitive impairment identified in the Umeed sample had no effect upon attendance at post-test counselling or linkage to care may suggest that the deficits identified were not severe enough to impact upon participants’ ability to understand and retain information given at time of testing, or to inhibit their ability to plan and execute their return visit.

The quantitative study design of Umeed and the fact that we measured mental health at a single time-point means that the mechanisms for the relationship between psychological factors and service use outcomes are unclear. Further observational, qualitative research is necessary to explore how mental health fits into the complex networks of concerns (stigma, illness/treatment beliefs etc.) that have been found to influence treatment-related behaviours and outcomes [36,37].

## Conclusions

As the gateway to assessment and treatment, receiving a diagnosis at post-test counselling is an essential component of the pathway to care. Our findings highlight the potential importance of CMD and AUD in linkage to care for HIV/AIDS. In particular, we found strong evidence to suggest that CMD may inhibit attendance for post-test counselling. Previous analysis of the Umeed dataset suggested that impaired cognitive functioning and CMD are associated with HIV seropositive status, prior to participant’s knowledge of the test result - we suggested that shared risk factors or underlying neurological damage due to HIV may account for this finding [34]. Exploratory post-hoc analyses suggest that sub-threshold symptoms of depression and anxiety at the time of testing may decrease the probability of people living with HIV/AIDS taking the next step in accessing specialist care for the disease. It will be important to carry out further prospective research to examine CMD symptoms over time with the aim of identifying periods of greatest risk, both in terms of severity of CMD symptoms and the potential adverse impact of this morbidity upon HIV outcomes. Our findings suggest the need to move towards a broader conceptualisation of the interrelationship between mental health and HIV wherein the possible impact of symptoms of depression and anxiety and the trajectory of mental health over time are considered at every stage of the continuum of care, from testing to lifelong adherence. If the Umeed study findings are replicated elsewhere, it may be important to consider the development and evaluation of early brief psychological interventions for all those with symptoms of common mental disorders, in order to improve the mental health and HIV outcomes (linkage with care and downstream clinical outcomes) of people newly diagnosed with HIV/AIDS.

## Competing interests

The authors declare that they have no competing interests.

## Authors’ contributions

MP & RM conceived of the study and planned analyses. RM carried out analyses. VP & MA participated in the design of the study. RM & PK co-ordinated study staff and data collection. SR provided provided on-site supervision during data collection. RP advised on study design and ethical considerations. All authors read and approved the final manuscript.

## Pre-publication history

The pre-publication history for this paper can be accessed here:

http://www.biomedcentral.com/1471-244X/14/188/prepub

## Supplementary Material

Additional file 1 Post-hoc analyses. Click here for file
