# Supplementary material for: Psychological predictors for attendance of post-HIV test counselling and linkage to care: the Umeed cohort study in Goa, India
Source: BMC Psychiatry. 2014 Jun 30;14:188. doi: 10.1186/1471-244X-14-188 (PMC4083128; doi:10.1186/1471-244X-14-188)
Supplement: Additional file 1 — Post-hoc analyses. [file 1471-244X-14-188-S1.docx]

| Additional file1: Table S1. Sociodemographic characteristics of refusals and participants | | | | | | |
| --- | --- | --- | --- | --- | --- | --- |
|  | Refusals | | Participants | | Chi-squared (degrees of freedom) | *p-value |
|  | N | % | N | % |  |  |
| Total | 315 | 13.82 | 1964 | 86.18 |  |  |
| Total with data | 291 | 12.77 | 1934 | 84.86 |  |  |
| Sex |  |  |  |  | 0.71 (1) | 0.40 |
| Male | 130 | 44.67 | 915 | 47.43 |  |  |
| Female | 161 | 55.33 | 1014 | 52.57 |  |  |
| Age (years) |  |  |  |  | 8.84 (6) | 0.18 |
| 18-25 | 56 | 19.24 | 444 | 22.96 |  |  |
| 26-30 | 46 | 15.81 | 368 | 19.03 |  |  |
| 31-35 | 50 | 17.18 | 307 | 15.87 |  |  |
| 36-40 | 42 | 14.43 | 299 | 15.46 |  |  |
| 41-45 | 37 | 12.71 | 168 | 8.69 |  |  |
| 46-50 | 26 | 8.93 | 155 | 8.01 |  |  |
| >51 | 34 | 11.68 | 193 | 9.98 |  |  |
| Ethnicity |  |  |  |  | 33.47 (1) | <0.01 |
| Goan | 215 | 73.88 | 1082 | 55.95 |  |  |
| Non-Goan | 76 | 26.12 | 852 | 44.05 |  |  |
| Education |  |  |  |  | 161.96 (3) | <0.01 |
| No education | 20 | 7.12 | 350 | 18.10 |  |  |
| Primary | 174 | 61.92 | 485 | 25.08 |  |  |
| Secondary | 80 | 28.47 | 941 | 48.66 |  |  |
| Higher education | 7 | 2.49 | 158 | 8.17 |  |  |
| Religion |  |  |  |  | 25.01 (3) | <0.01 |
| Hindu | 197 | 67.93 | 1406 | 72.70 |  |  |
| Christian | 78 | 26.90 | 316 | 16.34 |  |  |
| Muslim | 15 | 5.17 | 209 | 10.81 |  |  |
| Sikh | 0 | 0.00 | 1 | 0.05 |  |  |
| Other | 0 | 0.00 | 2 | 0.10 |  |  |
| * Differences between refusals and participants were tested with the Pearson chi-square test for homogeneity | | | | | | |

| **Additional file1: Table S2. Bivariate and multivariate analysis of association of PHQ-9 and GAD score categories with attendance of ART Centre** | | | | | | |
| --- | --- | --- | --- | --- | --- | --- |
| PHQ-9 score category | Prevalence among HIV+ve PTC attendees N(%) | Attendance among exposure category N(%) | Unadjusted odds ratio with 95% CI | P-value | Adjusted* odds ratio with 95% CI | P-value |
| 0 | 45 (24.6) | 39 (86.7) | ref |  | ref |  |
| 1-4 | 93 (50.8) | 58 (62.4) | 0.25 (0.09-0.69) | 0.01 | 0.25 (0.09-0.69) | 0.01 |
| 5-9 | 33 (18.0) | 19 (57.6) | 0.21 (0.06-0.68) | 0.01 | 0.18 (0.06-0.57) | <0.01 |
| >10 | 12 (6.6) | 8 (66.7) | 0.31 (0.07-1.41) | 0.12 | 0.19 (0.04-0.92) | 0.04 |
| GAD-7 score category |  |  |  |  |  |  |
| 0 | 43 (23.5) | 35 (81.4) | ref |  | ref |  |
| 1-4 | 117 (63.9) | 78 (66.7) | 0.46 (0.19-1.09) | 0.07 | 0.42 (0.17-1.04) | 0.06 |
| 5-9 | 20 (10.9) | 10 (50.0) | 0.23 (0.07-0.79) | 0.01 | 0.16 (0.05-0.57) | <0.01 |
| >10 | 3 (1.6) | 1 (33.3) | 0.11 (0.01-1.63) | 0.05 | 0.09 (0.01-1.23) | 0.07 |
| *Adjusted for sex, age, marital status, economically active (y/n) | | | | | | |

| **Additional file1: Table S3**  **Bivariate & multivariate analysis of association of PHQ-9 score category with attendance for post-test counselling** | | | | | | |
| --- | --- | --- | --- | --- | --- | --- |
| PHQ-9 score category | Prevalence among ART Centre attendees N(%) | Attendance among exposure category N(%) | Unadjusted odds ratio with 95% CI | P-value | Adjusted* odds ratio with 95% CI | P-value |
| 0 | 561 (29.0) | 497 (88.6) | ref |  | ref |  |
| 1-4 | 982 (50.8) | 857 (87.3) | 0.88 (0.64-1.23) | 0.45 | 0.89 (0.64-1.23) | 0.47 |
| 5-9 | 311 (16.1) | 271 (87.1) | 0.87 (0.57-1.33) | 0.53 | 0.90 (0.59-1.39) | 0.64 |
| >10 | 80 (4.1) | 59 (73.8) | 0.36 (0.20-0.64) | <0.01 | 0.39 (0.22-0.70) | <0.01 |
| GAD-7 score category |  |  |  |  |  |  |
| 0 | 479 (24.8) | 421 (87.9) | ref |  | ref |  |
| 1-4 | 1230 (63.6) | 1081 (87.9) | 1.00 (0.72-1.38) | 1.00 | 1.02 (0.74-1.42) | 0.88 |
| 5-9 | 204 (10.6) | 168 (82.4) | 0.64 (0.41-1.01) | 0.06 | 0.65 (0.41-1.03) | 0.07 |
| >10 | 21 (1.1) | 14 (66.7) | 0.28 (0.11-0.71) | 0.01 | 0.29 (0.11-0.76) | 0.01 |
| *Adjusted for sex, HIV status, hazardous alcohol use, verbal fluency, delayed recall | | | | | | |
